# Bacterial Diversity and Nitrogen Utilization Strategies in the Upper Layer of the Northwestern Pacific Ocean

**DOI:** 10.3389/fmicb.2018.00797

**Published:** 2018-04-25

**Authors:** Yuan-Yuan Li, Xiao-Huang Chen, Zhang-Xian Xie, Dong-Xu Li, Peng-Fei Wu, Ling-Fen Kong, Lin Lin, Shuh-Ji Kao, Da-Zhi Wang

**Affiliations:** ^1^State Key Laboratory of Marine Environmental Science, College of the Environment and Ecology, Xiamen University, Xiamen, China; ^2^State Key Laboratory of Marine Environmental Science, College of Ocean and Earth Sciences, Xiamen University, Xiamen, China

**Keywords:** Northwestern Pacific Ocean, bacterial diversity, nitrogen utilization genes, urea, cyanobacteria

## Abstract

Nitrogen (N) is a primary limiting nutrient for bacterial growth and productivity in the ocean. To better understand bacterial community and their N utilization strategy in different N regimes of the ocean, we examined bacterial diversity, diazotrophic diversity, and N utilization gene expressions in the northwestern Pacific Ocean (NWPO) using a combination of high-throughput sequencing and real-time qPCR methods. 521 and 204 different operational taxonomic units (OTUs) were identified in the 16s rRNA and nifH libraries from nine surface samples. Of the 16s rRNA gene OTUs, 11.9% were observed in all samples while 3.5 and 15.9% were detected only in N-sufficient and N-deficient samples. *Proteobacteria, Cyanobacteria* and *Bacteroidetes* dominated the bacterial community. *Prochlorococcus* and *Pseudoalteromonas* were the most abundant at the genus level in N-deficient regimes, while *SAR86, Synechococcus* and *SAR92* were predominant in the Kuroshio-Oyashio confluence region. The distribution of the nifH gene presented great divergence among sampling stations: *Cyanobacterium_UCYN-A* dominated the N-deficient stations, while clusters related to the *Alpha-, Beta-*, and *Gamma-Proteobacteria* were abundant in other stations. Temperature was the main factor that determined bacterial community structure and diversity while concentration of NO_X_-N was significantly correlated with structure and distribution of N_2_-fixing microorganisms. Expression of the *ammonium transporter* was much higher than that of *urea transporter subunit A* (*urtA*) and *ferredoxin-nitrate reductase*, while *urtA* had an increased expression in N-deficient surface water. The predicted ammonium transporter and ammonium assimilation enzymes were most abundant in surface samples while urease and nitrogenase were more abundant in the N-deficient regions. These findings underscore the fact that marine bacteria have evolved diverse N utilization strategies to adapt to different N habitats, and that urea metabolism is of vital ecological importance in N-deficient regimes.

## Introduction

Nitrogen (N) is an essential macronutrient for all organisms in the ocean and the availability of nitrogenous nutrients regulates biological productivity in most marine systems, and subsequently influences carbon cycling in the ocean (Zehr and Ward, 2002). The N cycle is regulated by complex microbial processes, such as N_2_ fixation, ammonia oxidation, nitrification, and de-nitrification (Voss et al., 2013). Thus, deciphering the relationship between microbial diversity and N utilization strategies is essential to reveal the oceanic N cycle (Giovannoni and Vergin, 2012; Bowen et al., 2014).

In coastal and upwelling areas, inorganic N nutrients are usually sufficient to support microbial growth. Dissolved inorganic N (DIN), such as ${\text{NO}}_{3}^{-}$ or ${\text{NO}}_{2}^{-}$, is reduced to ammonium (${\text{NH}}_{4}^{+}$), and then enters the central N assimilation pathway known as the glutamine synthetase/glutamate synthase cycle (Casciotti, 2016). However, most tropic and subtropic oceans are oligotrophic with undetected DIN (Zehr and Kudela, 2011). Much effort has been devoted to exploring the adaptive mechanisms of microorganisms to N-deficient conditions. One of the efficient ways is to increase the gene expressions of high-affinity transporters and utilize various forms of dissolved organic N (DON) (Kent et al., 2016). For example, many *Prochlorococcus* genes for the transport and assimilation of N, including *amt1, urt*, and *cyn* (cyanate genes) are up-regulated during N starvation (Tolonen et al., 2006). Urea transporter is abundant in oligotrophic surface oceans (Wang et al., 2011; Saito et al., 2014), suggesting that urea is an important N source for microorganisms in N limited waters. In the open ocean, urea is usually regarded as the by-product of cell metabolism or dead cell decomposition. Cyanobacteria and heterotrophic bacteria are both producers of urea, while the cyanobacterial genera *Prochlorococcus* and *Synechococcus* are the most prevalent ureolytic planktonic microorganisms in subtropic and tropic oligotrophic oceans (Collier et al., 2009). They are capable of absorbing and then decomposing urea into ${\text{NH}}_{4}^{+}$ through the urea transporter and urease (Solomon et al., 2010; Christie-Oleza et al., 2015).

Biological N_2_ fixation by diazotrophs is another important strategy for microorganisms to survive in N-deficient waters (Montoya et al., 2004). N_2_ absorption into the cells is catalyzed by nitrogenase, and the *nifH* gene is widely used as a biomarker to study the diversity of N_2_-fixing bacteria in the ocean (Gaby and Buckley, 2014). For years, *Trichodesmium* has been considered as the major N_2_ fixer in the ocean and much effort has been devoted to the distribution and functioning of *Trichodesmium* populations in the global ocean (Goebel et al., 2010; Jayakumar et al., 2012; Rouco et al., 2016). However, recent studies demonstrate that unicellular diazotrophic cyanobacteria and non-cyanobacterial diazotrophs are widely distributed in the ocean, and unicellular cyanobacteria contribute significantly to the oceanic N fixation in oligotrophic waters (Zehr et al., 2001; Farnelid et al., 2011; Karl and Church, 2014).

The northwestern Pacific Ocean (NWPO), as one of the world's largest oligotrophic regions (Polovina et al., 2008), represents the largest oceanic currents on Earth but remains largely unexplored regarding N cycling mediated by microbes. Marine bacterial diversity, particularly from the perspective of N-cycling, is important to investigate, since N is often a primary limiting nutrient for marine microbial growth/metabolism (Zehr and Kudela, 2011). In our study, we investigated bacterial diversity, and multiple N-related genes, including *urea transporter subunit A* (*urtA*), *ferredoxin-nitrate reductase* (*narB*), and *ammonium transporter* (*amt1*), are examined and interpreted alongside 16s rRNA diversity in what appears to be an under-sampled region of the NWPO. Moreover, special attention was paid to the unicellular diazotrophs in the North Pacific Ocean. The goal of our study was to unveil the dominant bacterial groups and their N utilization strategies in different N regimes of the NWPO.

## Materials and methods

### Study area and sample collection

**Figure 1:**
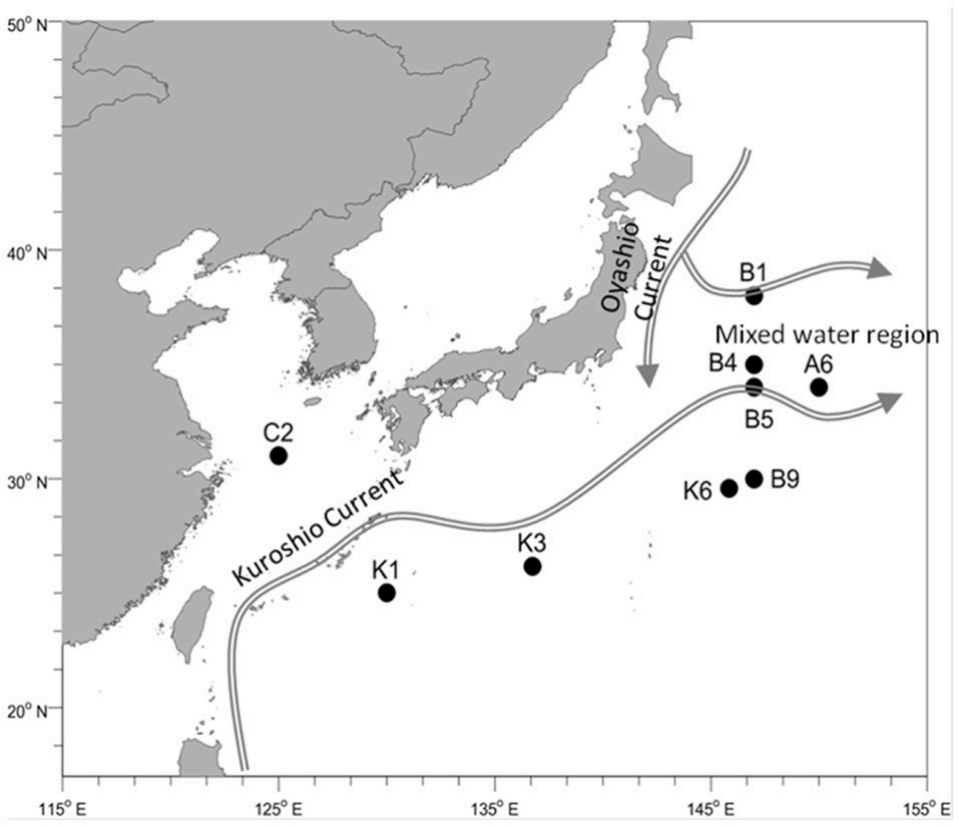
Sampling locations and ocean currents in the northwestern Pacific Ocean.

The NWPO cruise was conducted from 30th March to 10th May 2015, crossing through the East China Sea and pelagic western Pacific Ocean (Figure 1). Microbial samples of surface seawater (0–5 m) were collected from nine stations for bacterial diversity analysis. At each sampling station, approximately 30 L of seawater was collected using Niskin bottles attached to a CTD rosette, and pre-filtered through a 3 μm membrane (Whatman 47 mm polycarbonate membrane) to remove large zooplanktonas well as separate or fasciculate filamentous *Trichodesmium*. Then, the seawater was filtered through a 0.2 μm membrane (Whatman 47 mm polycarbonate membrane) to concentratethe microorganisms. Microbial samples for N-utilizing gene analysis were collected from both the surface and deep chlorophyll maximum (DCM) layers and 30 L of seawater for each sample was filtered as mentioned above. It should be noted that a water sample at the 20-m depth was collected from station C2 because of the well-mixed water column, while only surface waters were sampled from stations K6 and B5 owing to poor sea conditions. Collected samples were immediately frozen in liquid nitrogen after 20 min processing time and stored at −80°C before further analysis. Physicochemical parameters of the surface and DCM layers in each station were analyzed. Temperature, salinity, and Chlorophyll a (Chla) were measured using a conductivity-temperature-depth rosette system [CTD, Sea Bird Electronics] (Data Sheet 2). Skalar San++ continuous flow analyzer was used to measure the concentrations of phosphate, silicic acid, ammonium, and nitrate plus nitrite (Gordon et al., 1993). Nitrate concentration was determined by subtracting nitrite concentration from the total concentration of nitrite and nitrate.

### Nucleic acid extraction and cDNA synthesis

Prior to nucleic acid extraction, the samples were thawed on ice. DNA extraction was performed following the instruction manual of the PowerSoil® DNA Isolation Kit (Cat. No. 12888, MOBIOLaboratories, USA) while three biologically repeated RNA samples were purified as described by Atshan et al. (2012) with a few modifications. Briefly, each thawed sample was lysed in Trizol reagent (1 mL, Life Technologies) with beads beating in a homogenizer (06404-200-RD000, Bertin Minilys) for 5 min. Then, the cell lysate was transferred into a new sterile tube with the addition of 200 μL chloroform, and centrifuged at 12,000 g for at least 15 min at 4°C. The resulting supernatant was purified using the RNeasy® Mini Kit (Cat. No. 74104, Qiagen, Germany). Both DNA and RNA solutions were dissolved in DEPC-treated water and quantified using a NanoVue Plus spectrophotometer (GE Life sciences). The procedure for environmental cDNA synthesis was immediately carried out following the instruction manual of the QuantiTect® Reverse Transcription Kit (Cat. No. 205311, Qiagen, Germany). Briefly, gDNase (provided in the Kit) was used to remove genomic DNA, then 10 μL of pure RNA template was pooled with random primers, reverse transcriptase and the dNTP mixture. Reverse transcription was conducted at 42°C for 25 min and 95°C for 3 min. The cDNA samples were maintained at −20°C.

### Sequencing of 16s-rRNA and nifH genes

The V3 region of 16s rRNA in each surface DNA sample was amplified using the bacterial specific primers 338F: 5′- barcode-ACTCCTACGGGAGGC AGCA-3′, and 806R: 5′- GGACTACHVGGGTWTCTAAT-3′ (Mori et al., 2014), where the barcode is an eight-base sequence unique to each sample. The PCR reaction was performed in triplicate 20 μL mixtures containing 10 ng of template DNA, 0.2 μM primers, 0.1 mM dNTPs, 0.4 μL FastPfu Polymerase, 5x FastPfu buffer, and 0.2 μL BSA based on the TransGen AP221-02 kit. The cycling protocol was: 95°C for 3 min, 27 cycles of 95°C for 30 s, 55°C for 30 s, 72°C for 45 s, and finally, 72°C for 10 min to ensure a complete extension. The nifH genes in all DNA extracts were amplified using the targeted primers, nifH-F: 5′-AAAGGYGGWATCGGYAARTCCACCAC-3′ and nifH-R: 5′- TTGTTSGCSGCRTACATSGCCATCAT-3′) as used by Török and Kondorosi (1981). The cycling conditions and post processing were as above except that the cycle number was increased to 35. Amplicons were extracted from 2% agarose gels and purified using the AxyPrep DNA Gel Extraction Kit (Axygen Biosciences, Union City, CA, USA) in accordance with the manufacturer's instructions and quantified using QuantiFluor™ -ST (Promega, USA). Purified amplicons were pooled in equimolar and paired-end sequences (2 × 250) on an Illumina MiSeq platform following the standard protocol.

Raw reads were de-multiplexed, quality-filtered using QIIME (version 1.9.1) with the following criteria: (i) 300 bp reads were truncated at any site receiving an average quality score <20 over a 50 bp sliding window, discarding the truncated reads that were shorter than 50 bp; (ii) exact barcode matching, two nucleotide mismatch in primer matching, reads containing ambiguous characters were removed; and (iii) only sequences that overlapped by more than 10 bp were assembled,based on their overlap sequence. Reads which could not be assembled were discarded. Operational taxonomic units (OTUs) were clustered with 99% similarity cutoff using UPARSE (version 7.1 http://drive5.com/uparse/) and chimeric sequences were identified and removed using UCHIME. The taxonomy of each 16s rRNA gene sequence was mapped to the SILVA rRNA database (Release119, http://www.arb-silva.de) (Quast et al., 2013), while the nifH nucleic acid sequences were aligned to the FunGene database derived from GenBank (Release 7.3, http://fungene.cme.msu.edu/) (Fish et al., 2013).

The full 16s rRNA gene sequences of nine libraries and nifH gene sequences of seven libraries were deposited in GenBank under BioProjectID PRJNA434503. The individual accession numbers SAMN08563407-08563415 represented 16s rRNA libraries and SAMN08563568-08563574 represented nifH libraries.

### Cyanobacterial specific primer design and validation

Considering the ecological importance of the cyanobacteria *Prochlorococcus* and *Synechococcus* in the ocean, specific primers *urtA* (F: 5′-CWGGWCCYTGWGGRGCATCRA-3′, R: 5′-ACTAYGGYGCTT GGAACTACAT-3′; position 189–420 bp), *amt1* (F: 5′-ACCTGYTGGATTGCYTGGTCT-3′, R: 5′- AAGTCKGGATAWGCYTCCAT-3′; position 1,340–1,479 bp), and the *Synechococcus* specific primer *narB* (F: 5′-TGGCAGCAGRTSGAAGCGATG-3′, R: 5′-AGGCCTCGCTCACCACCACCA-3′; position 1,200–1,342 bp) were designed based on the database of the Cyanobacterial Knowledge Base (http://nfmc.res.in/ckb) (Peter et al., 2015). To minimize sampling or processing differences between samples, specific cyanobacteria 16s rRNA primers (16SCF: 5′-GGCAGCAGTGGGGAATTT TC-3′, 16SUR: 5′-GTMTTACCGCGGCTG CTGG-3′) were applied as internal control genes (Kyoung-Hee et al., 2012).

Sequences of targeted genes in diverse species were downloaded and blasted, but only highly conserved regions among ecotypes were conveyed to the online Primer Designing Tool (https://www.ncbi.nlm.nih.gov/tools/primer-blast/), while the melting temperature threshold was settled at 60°C. To verify the effectiveness and specificity of primers, PCR products were separated on agarose gels, ensuring that only the desired lengths of segments were amplified. Furthermore, PCR products were cloned into T-vector (Takara) and randomly 35 clones from each primer set were sequenced. Sequences obtained were blasted in the National Center for Biotechnology Information gene database (Brown et al., 2015). Only primers with at least 25 positive clones with identity > 90% and *E*-value < 0.01 were chosen (Bayer et al., 2014).

### Real-time qPCR assay

The real-time qPCR reaction was carried out in a volume of 20 μL containing 10 μL of 2 × SuperReal PreMix Plus (Cat. No. 208054, Qiagen,Germany), 0.7 μL of each primer (0.4 μmol/L final concentration), 1.5 μL cDNA (total mass between 10 and 30 ng), 0.4 μL 50 × ROX Reference Dye provided in the Qiagen kit, and 6.6 μL sterilized H_2_O. For each PCR plate, three negative controls without template cDNA were installed. Real-time qPCR assay was performed in a Thermal Cycler, an ABI 7500 instrument with an initial stage of preheating at 95°C for 10 min, followed by 40 cycles of 95°C for 15 s and 60°C for 1 min. Representative samples of each station with specific primers were biologically repeated three times. The melting curve procedure was initiated consecutively, under conditions of 95°C for 15 s, 60°C for 1 min, and then gradually increasing to 95°C. The specific cyanobacterial 16s rRNA gene was applied as the reference gene to normalize cell numbers and random errors between samples.

qPCR results were analyzed using matched 7500 software (v1.3.1). The threshold cycle numbers (Ct value) and baseline were automatically determined using the software, making sure that amplification curves initiated after the maximum cardinality and Ct values fell exactly on the exponential growth period of the amplification curve. Gene transcript (RT) was calculated and normalized using the internal control cyanobacterial 16s rRNA gene as in the equations below:

(1) $$\begin{array}{r} RT=2^{-\Delta Ct}\\ \Delta Ct=C_{\text{t}}-C_{16\text{s}} \end{array}$$

where Ct is the cycle number of the target gene, and C_16s_ refers to the cycle number of 16s rRNA within the same template.

### PICRUSt and other statistical analyses

The 16s rRNA data were analyzed using PICRUSt genome prediction software (v0.9.2) (Langille et al., 2013). OTUs assigned at 99% similarity were mapped to the Greengenes database (v13.5) for functional prediction, with normalization to control differences in 16S rRNA copy number among OTUs. Functional predictions were assigned to EggNOG database (v4.0) for all genes.

Two diversity indices, Chao1 index (Chao, 1984) and Shannon index (Hill et al., 2003), were used to compare bacterial and diazotrophic alpha diversity based on Mothur software (v.1.30.1) (Schloss et al., 2011). Non-metric multidimensional scaling (NMDS) (Rivas et al., 2013) and Principal co-ordinates analysis (PCoA) were used to analyze beta diversity in different treatments, which was performed on the basis of the calculated Bray-Curtis distance. Correlation between environmental variables and bacterial community structure (Bray-Curtis distance) was estimated using Mantel test in R software (v3.4.3, vegan package). Spearman's correlation coefficient was used to investigate the possible correlation between the relative abundances of bacteria and environmental variables (Hauke and Kossowski, 2011). The Spearman's correlation coefficients were calculated using IBM Predictive Analytics Software (PASW) Statistics (v18), and the results were subject to *t*-test for significance. The heatmap was constructed using the R package “pheatmap.”

## Results

### Overview of physicochemical parameters in the survey area

Temperature varied among different sampling stations, ranging from 13.6 to 23.3°C, but no difference was observed between the surface and DCM layers in each station (Table 1). Salinity was very stable and varied only slightly among the different sampling stations, except for the coastal station C2, which was characterized by the lowest salinity. The surface seawater of the transect K stations was characterized by lower concentrations of DIN (${\text{NO}}_{2}^{-}$, ${\text{NO}}_{3}^{-}$, ${\text{NH}}_{4}^{+}$) while the concentration of NO_X_ was below 0.1 μmol/L and ammonium fluctuated between 0 and100 nmol/L. With the flow direction of the warm Kuroshio Current, concentrations of DIN increased gradually and peaked in the northern stations B4 and B1. Concentration of chl*a* presented a similar pattern. Concentrations of phosphorus were low in the survey area, ranging between 0.01 and 0.42 μmol/L, and concentrations of silicate were high in stations C2 and B9 (Table 1).

**Table 1:** Sampling sites and physical-chemical parameters.

**Station**	**Date (m/d/y)**	**Latitude (°N)**	**Longitude (°E)**	**Depth (m)**	**Temperature (°C)**	**Salinity (PSU)**	**Chla. (μg/L)**	**${\text{PO}}_{4}^{3-}$(μM)**	**${\text{SiO}}_{3}^{2-}$(μM)**	**${\text{NO}}_{2}^{-}$(μM)**	**${\text{NO}}_{3}^{-}$(μM)**	**${\text{NH}}_{4}^{+}$ (μM)**
C2	5/3/2015	31.00	125.00	20	14.6	32.9	2.45	0.21	11.34	0.32	1.60	–
K1	4/4/2015	25.00	130.00	5	23.2	34.89	0.01	–	–	–	–	–
				60	22.0	34.87	0.93	0.01	1.18	0.03	0.07	0.030
K3	4/7/2015	26.18	136.73	5	23.3	34.77	0.01	0.01	0.32	0.01	0.08	0.032
				90	20.0	34.87	0.52	0.14	1.32	0.03	1.71	0.029
K6	4/9/2015	29.57	145.84	5	20.6	34.93	0.07	0.01	2.79	0.01	0.04	0.115
B9	4/10/2015	30.00	147.00	5	20.3	34.82	0.06	0.14	22.38	0.01	0.13	0.059
				55	18.0	34.83	0.66	0.13	20.90	0.01	0.12	0.078
B5	4/13/2015	34.00	147.00	5	17.4	34.77	0.67	0.03	2.44	–	0.30	–
A6	4/23/2015	34.00	150.00	5	17.7	34.86	1.24	0.07	2.95	0.02	0.17	0.119
				45	17.4	34.77	2.30	0.12	2.52	0.12	0.73	0.213
B4	4/27/2015	35.00	147.00	5	20.5	34.84	1.03	0.02	6.80	0.38	5.40	–
				40	19.9	34.83	0.17	0.04	0.57	0.45	0.16	–
B1	4/25/2015	38.00	147.00	5	13.7	34.08	1.92	0.42	7.94	0.20	4.83	0.207
				30	13.6	34.43	2.11	0.41	7.91	0.21	4.81	0.205

*PSU, practical salinity unit; Chla, chlorophyll a. “–” means the value is below the detectionlimit*.

### Bacterial diversity in the surface waters of the NWPO

16s rRNA libraries of nine surface water samples collected from the NWPO were constructed to determine microbial community composition and abundance. A total of 261,896 qualified reads (ranging from 21,614 to 39,100 per DNA sample) were recovered from 129 Mb bases and were clustered into 521 different OTUs (average OTU number per sample, 263; average OTU length, 436 bp) with a 99% match (Table S1). Among them, 62 OTUs were found in all samples while 82 OTUs were unique to the N-deficient stations K1, K3, and K6 and 18 OTUs were unique to the N-sufficient stations B1 and B4.

**Figure 2:**
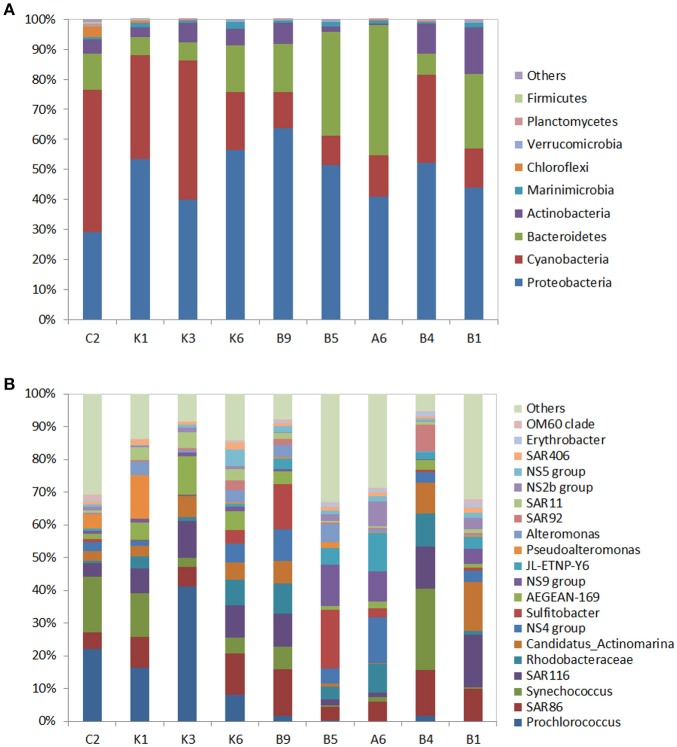
Phylogenetic composition of the 16s rRNA gene sequences in the surface water of the nine stations at the phylum level **(A)** and at the genus level **(B)**. “Others” refer to unclassified OTUs. In **(B)**, only the relative abundances of the top 20 genera are listed. For clarity, multiple OTUs with the same taxonomic classification were grouped together.

Totally, 261,896 reads were assigned to 15 phyla. The predominant phylum was the *Proteobacteria* (48.7% of total 16s rRNAgene sequences), followed by the *Cyanobacteria* (25.5%), *Bacteriodetes* (17.4%), and *Actinobacteria* (5.8%) (Figure 2A). These four phyla represented more than 96% of the bacterial community in each station. The *Alphaproteobacteria* (29.3%) and *Gammaproteobacteria* (21.7%) in the phylum *Proteobacteria*, were abundant in each station, but the genus compositions of these two classes presented differences among the stations. The most frequent phylotypes of *Gammaproteobacteria*, at genus level,were *SAR86, SAR92, Pseudoalteromonas*, and *JL-ETNP-Y6* (*Oceanospirilla*). The relative abundance of *SAR86* ranged between 0.5 and 2% of total bacteria. *Pseudoalteromonas* was more abundant in the oligotrophic station K1 (accountingfor 13.4% of the bacterial community) while *SAR92* and *JL-ETNP-Y6* presented the highest relative abundance in the stations B4 and A6, respectively. Within *Alphaproteobacteria*, the *Rhodospirillaceae (AEGEAN-169), SAR116*, and *SAR11* from *Alphaproteobacteria* were more abundant in the N-deficient K1, K3, and K6 stations (Figure 2B).

The *Cyanobacteria* were the second most prevalent group, and the proportion of *Cyanobacteria* was two-fold higher in the N-deficient stations (11.5%) than that in the N-sufficient stations (5.2%) (Figure 2A), indicating the strong competitive ability of the *Cyanobacteria* in the N-deficient ocean. Further classification at genus level revealed that *Prochlorococcus* dominated the N-deficient station K3 with 41.2% of the bacterial community; however, it declined to < 2% in the N-sufficient stations. Another cyanobacterial genus, *Synechococcus*, was widely distributed with a high proportion in the station B4, representing 24.9% of the bacterial community. *Flavobacteria*, the dominant subgroup of the *Bacteriodetes*, were also found in each sample, and accounted for 41.0% of the bacterial community in the station A6 (Figure 2B, Table S5).

**Figure 3:**
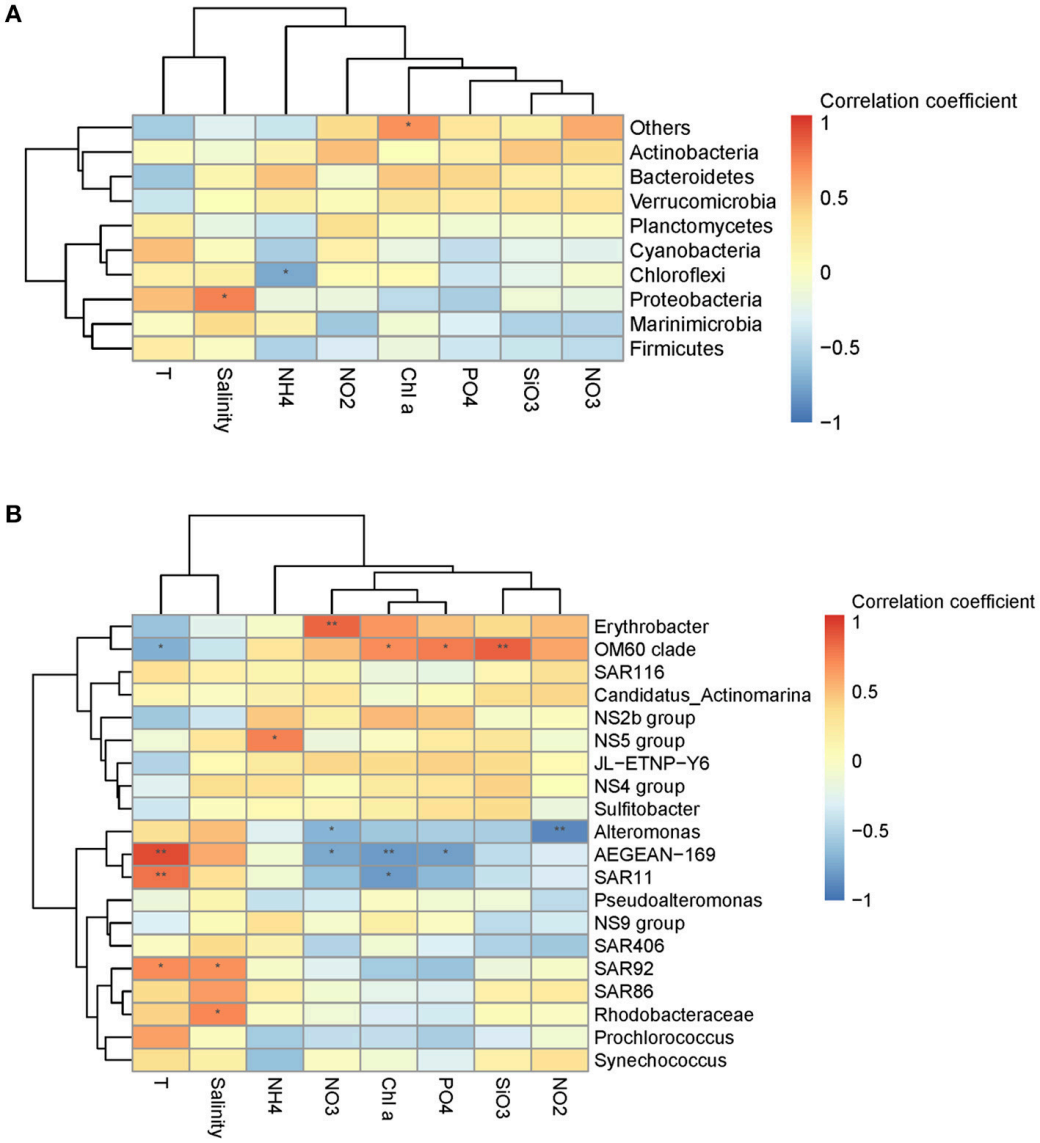
Spearman's rank correlation coefficient between relative abundance of bacterial communities and environmental variables at the phylum level **(A)** and the genus level **(B)**. **P < 0.01 and *P < 0.05 indicate significant correlation.

Mantel test analysis showed a significant positive correlation between bacterial community structure (Bray-Curtis distance) and the ambient temperature (Table 2). With regard to bacterial community diversity, NMDS and PCoA indexes presented positively correlated with temperature, but negatively correlated with concentrations of Chl*a* and phosphorus (Table 3; Table S2**)**. No significant correlation was observed between alpha bacterial diversity and community structure. Spearman correlation between bacterial community (OTU distributions of phylum and genus) and environmental variables was calculated based on PASW statistic. At the phylum level, distributions of *Proteobacteria* and *Cyanobacteria* showed weak correlations with both temperature and nutrients while distribution of *Chloroflexi* was negatively correlated with ammonium concentration (Figure 3A; Table S3**)**. At the genus level, temperature is negatively correlated with *OM60* clade, but positively correlated with *AEGEAN*−*169, SAR11*, and *SAR92* (Figure 3B; Table S3**)**. In addition, *Erythrobacter* was positively correlated with nitrate concentration while *AEGEAN*−*169* and *SAR11* showed negative correlations with concentrations of nitrate, Chl*a* and phosphorus. Only NS2b group was significantly positively correlated with ammonium concentration (Figure 3B; Table S3).

**Table 2:** The spearman's correlations among bacterial community, diazotrophic diversity, and environmental factors.

	**Spearman's correlation**							
		**T**	**Salinity**	**Chl a**.	**${\text{PO}}_{4}^{3-}$**	**${\text{SiO}}_{3}^{2-}$**	**${\text{NO}}_{2}^{-}$**	**${\text{NO}}_{3}^{-}$**	**${\text{NH}}_{4}^{+}$**
Bacterial diversity	OTUs	−0.350	−0.042	0.494	0.092	0.150	0.204	0.350	−0.557
	Chao1 estimator	−0.350	−0.042	0.494	0.092	0.150	0.204	0.350	−0.557
	Shannon index	−0.552	0.000	0.437	0.294	0.109	−0.188	0.050	0.144
	NMDS axis 1	0.933^**^	0.418	−0.795*	−0.837^**^	−0.533	−0.332	−0.633	−0.409
	PCoA axis 1	0.867^**^	0.427	−0.695^*^	−0.845^**^	−0.567	−0.306	−0.617	−0.514
Diazotrophic diversity	OTUs	−0.643	−0.396	0.667	0.559	0.357	0.318	0.857^*^	−0.729
	Chao1 estimator	−0.667	−0.427	0.709	0.600	0.414	0.406	0.883^**^	−0.736
	Shannon index	−0.571	−0.378	0.685	0.450	0.250	0.393	0.786^*^	−0.867^*^
	NMDS axis 1	−0.750	−0.288	0.829^*^	0.631	0.536	0.487	0.857^*^	−0.650
	PCoA axis 1	−0.750	−0.288	0.829^*^	0.631	0.536	0.487	0.857^*^	−0.650

** P < 0.01 and

* *P < 0.05 indicate significant correlation*.

**Table 3:** The spearman's correlations (*r*) between environmental factors and community structure (Bray-Curtis distance) determined by Mantel test.

	**Bacterial structure**		**Diazotrophic structure**	
	***r***	***P***	***r***	***P***
T	0.527^**^	0.004	0.339	0.100
Salinity	0.005	0.483	0.308	0.158
Chl.a	0.251	0.083	0.802^**^	0.005
${\text{PO}}_{4}^{3-}$	0.089	0.321	0.217	0.196
${\text{SiO}}_{3}^{2-}$	−0.172	0.798	−0.046	0.526
${\text{NO}}_{2}^{-}$	0.116	0.290	0.671^*^	0.025
${\text{NO}}_{3}^{-}$	0.235	0.116	0.816^**^	0.003
${\text{NH}}_{4}^{+}$	0.100	0.292	−0.082	0.553

** P < 0.01 and

* *P < 0.05 indicate significant correlation*.

### Distribution of N_2_-fixing bacteria

**Figure 4:**
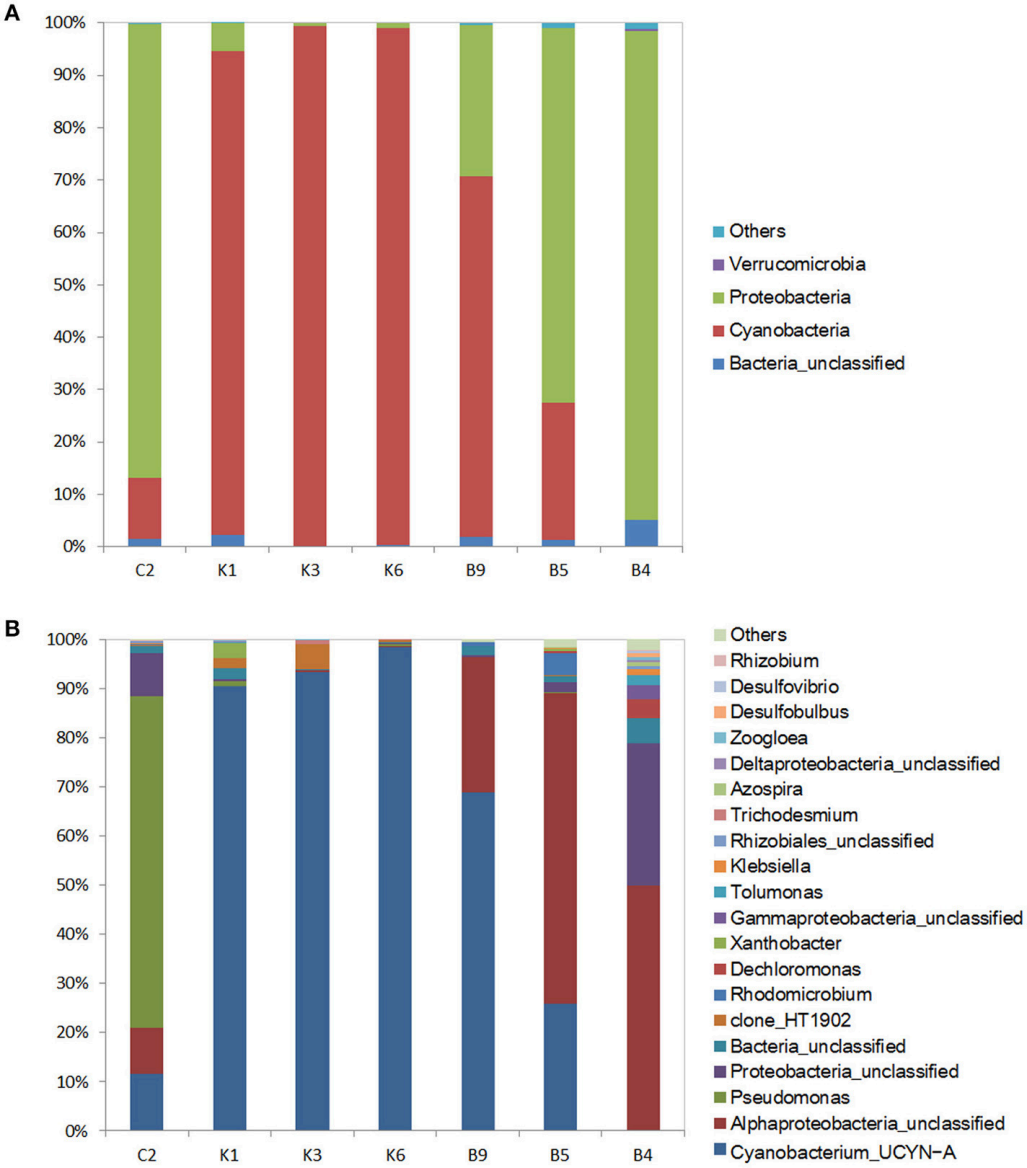
Phylogenetic composition of N_2_ fixing microorganisms in the surface of the seven stations at the phylum level **(A)** and the genus level **(B)**. “Others” represents the unclassified OTUs. In **(B)**, only relative abundances of the top 20 genera are listed.

In this study, the nifH gene was selected as the marker gene to identify unicellular diazotrophs in the NWPO. The nifH libraries were constructed using the same DNA samples as used for the16s rRNA libraries except for stations A6 and B1 since their nifH gene products were not detected using gel electrophoresis. Totally, 250,424 qualified sequences were grouped into 204 different OTUs (average OUT number per sample, 48; average read length, 450 bp) in seven stations (Table S1), and an average of 97.8% was affiliated with three phyla: the *Cyanobacteria* (60.5% of total *nifH* sequences), *Proteobacteria* (37.6%), and *Verrucomicrobia* (< 1%) (Figure 4A).

The *Cyanobacteria* were most abundant in the N-deficient K-transect (Figure 4A), comprising 92–99% of the diazotrophic community in N-deficient stations, and then decreased to 26.2% in station B5 and were nearly undetectable in the N-sufficient station B4. Further analysis at genus level showed that the majority of the *Cyanobacteria* OTUs belonged to *Cyanobacterium_UCYN–A* (*UCYN–A*) (Figure 4B), which was also found in the 16s rRNA libraries although it presented a low relative abundance to total sequences (<0.1%). In addition, the unicellular cyanobacterial *clone_HT1902* presented high abundance in the N-deficient station K3 (accounting for 5.2% of bacterial community). The second most abundant diazotroph was the *Proteobacteria*, but the proportions of alpha-, beta-, gamma-, and delta-proteobacterial subpopulations varied among the sampling stations. The gammaproteobacterial *Pseudomonadales* dominated in the coastal station C2, representing 67.5% of the diazotrophic community, while they were negligible in other stations. *Alphaproteobacteria* were more ubiquitous in transect B (19.6%) (Figure 4B).

Interestingly, the highest diversity of diazotrophs was observed in the station B4, where116 different OTUs were detected. Betaproteobacterial *Azospira*, and *Dechloromonas*, deltaproteobacterial *Desulfobulbus*, and *Desulfovibrio*, as well as gammaproteobacterial *Tolumonas* and *Klebsiella*, showed the highest relative abundances in the station B4. However, they were either insignificant or undetectable in other stations (Figure 4B). We were unable to classify approximately 1.7% of all qualified sequences and 4.8% of the proteobacterial sequences, because of the limitation of the database.

**Figure 5:**
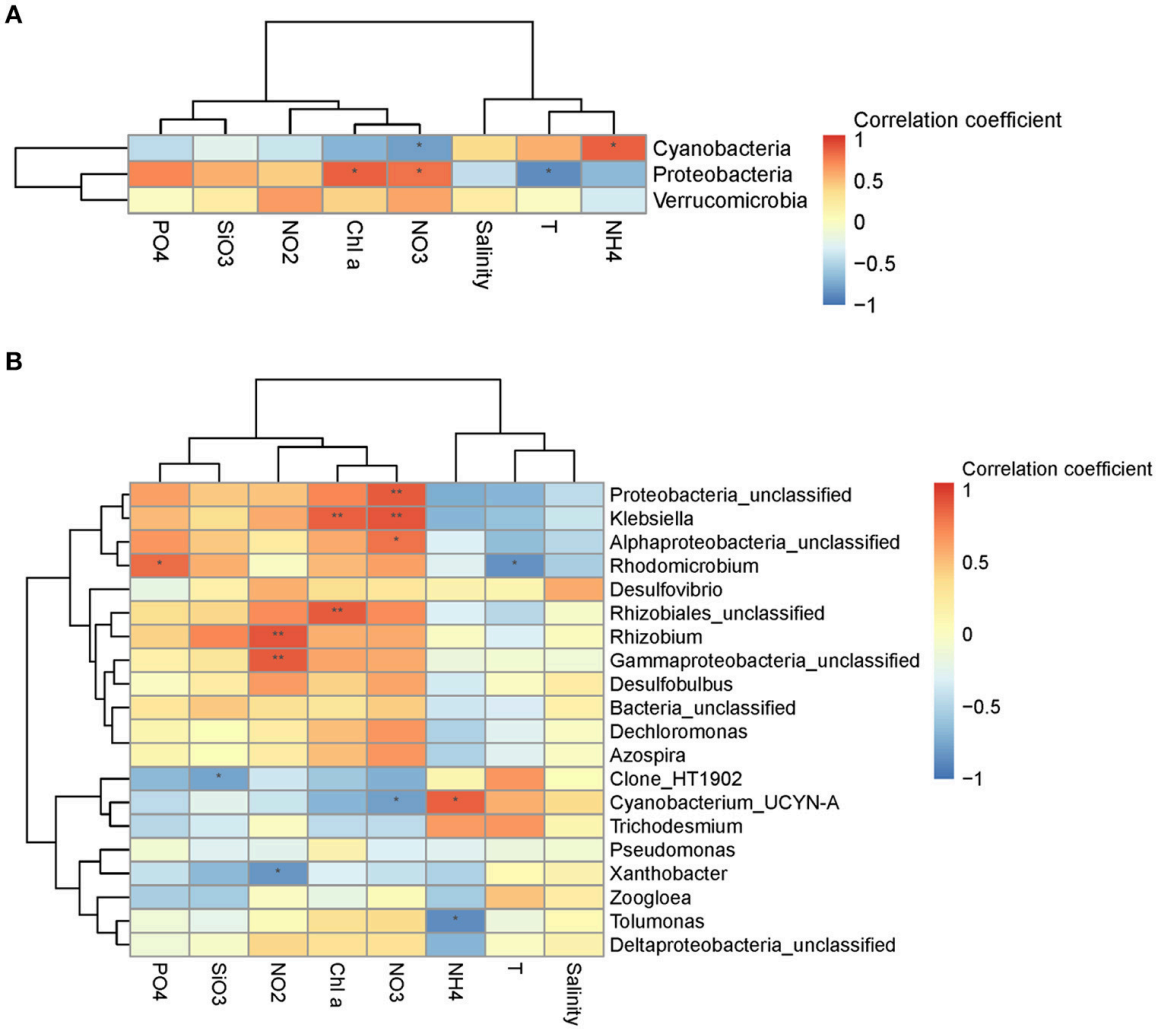
Spearman's rank correlation coefficient between relative abundance of diazotrophic communities and environmental variables at the phylum level **(A)** and the genus level **(B)**. **P < 0.01 and *P < 0.05 indicate significant correlation.

Diazotrophic community structure and beta-diversity were significantly positively correlated with concentrations of Chl*a* and nitrate, while alpha-diversity Shannon index was negatively correlated with ammonium concentration (Tables 2, 3; Table S2). Spearman analysis showed that *Cyanobacterium_UNCY-A* was positively correlated with ammonium concentration, but negatively correlated with nitrate concentration, while *Proteobacteria*, including *Klebsiella, Rhizobium* and large numbers of unclassified proteobacteria,were mostly positively correlated with concentrations of nitrate, nitrite and Chla (Figures 5A,B; Table S4).

### Expressions of key N-utilizing genes in *Prochlorococcus* and *Synechococcus*

**Figure 6:**
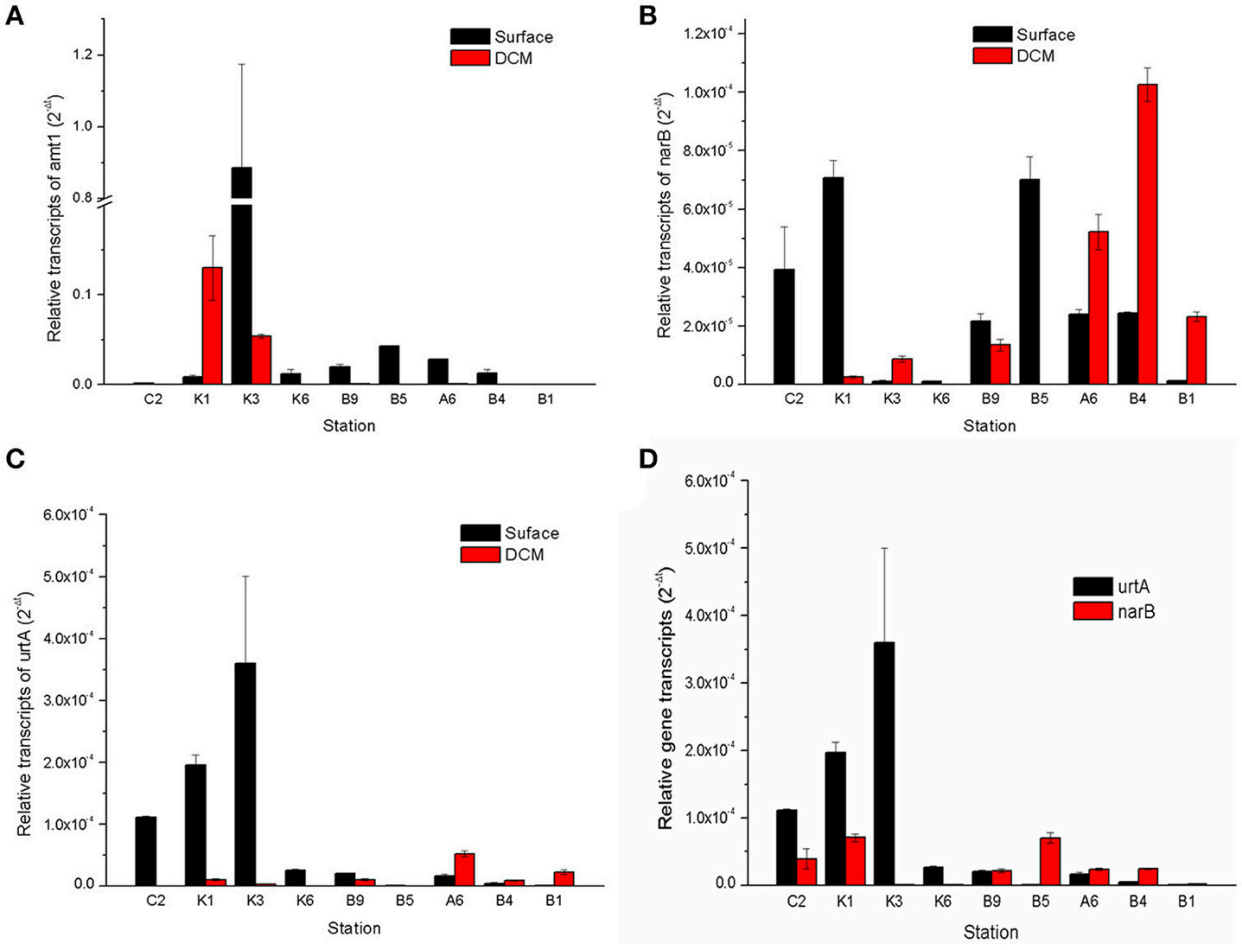
Relative transcripts of *amt1, narB*, and *urtA* in the cyanobacterial genera *Prochlorococcus* and *Synechococcus* between the surface and DCM layers of the sampling stations **(A–C)** and a comparison of the relative transcripts of *urtA* and *narB* in the surface samples **(D)**. Error bars represent the standard deviations of the valuesgenerated from three biological repeats.

Expressions of three key N-utilizing genes, *amt1, narB*, and *urtA* in *Prochlorococcus* and *Synechococcus* from both the surface and DCM layers were examined. Relative expression of the *amt1* gene was approximately two- to five-fold higher than that of the *narB* and *urtA* genes, especially in the N-deficient station K3 and the DCM layer of the station K1, but it decreased in the N-sufficient DCM layers (Figure 6A). Expression of the *narB* gene fluctuated in the surface layer among different stations, displaying no obvious change in different N regimes, but it was high in the DCM layers of stations A6, B4, and B1, and peaked in the N-sufficient station B4 (Figure 6B).

As well as ${\text{NH}}_{4}^{+}$ and nitrate, urea is another important source of regenerated N utilized by oceanic microorganisms (Berman and Bronk, 2003). In the surface layer, expression of the *urtA* gene increased gradually from the coastal station C2 to the pelagic stations K1, K2, and K3, and was about two- to three-fold higher than that of the *narB* gene in the N-deficient stations (Figure 6C), and it was very low in the station A6 and transect B. Moreover, different expression patterns of the *urtA* and *narB* genes were observed between the surface and DCM layers: expression of the *narB* gene was higher in the DCM layers, especially in the N-sufficient station B4; however, expression of the *urtA* gene was much higher in the surface layers of stations K1 and K3 than in the DCM layers (Figure 6D).

### Functional prediction of N-utilization genes

**Figure 7:**
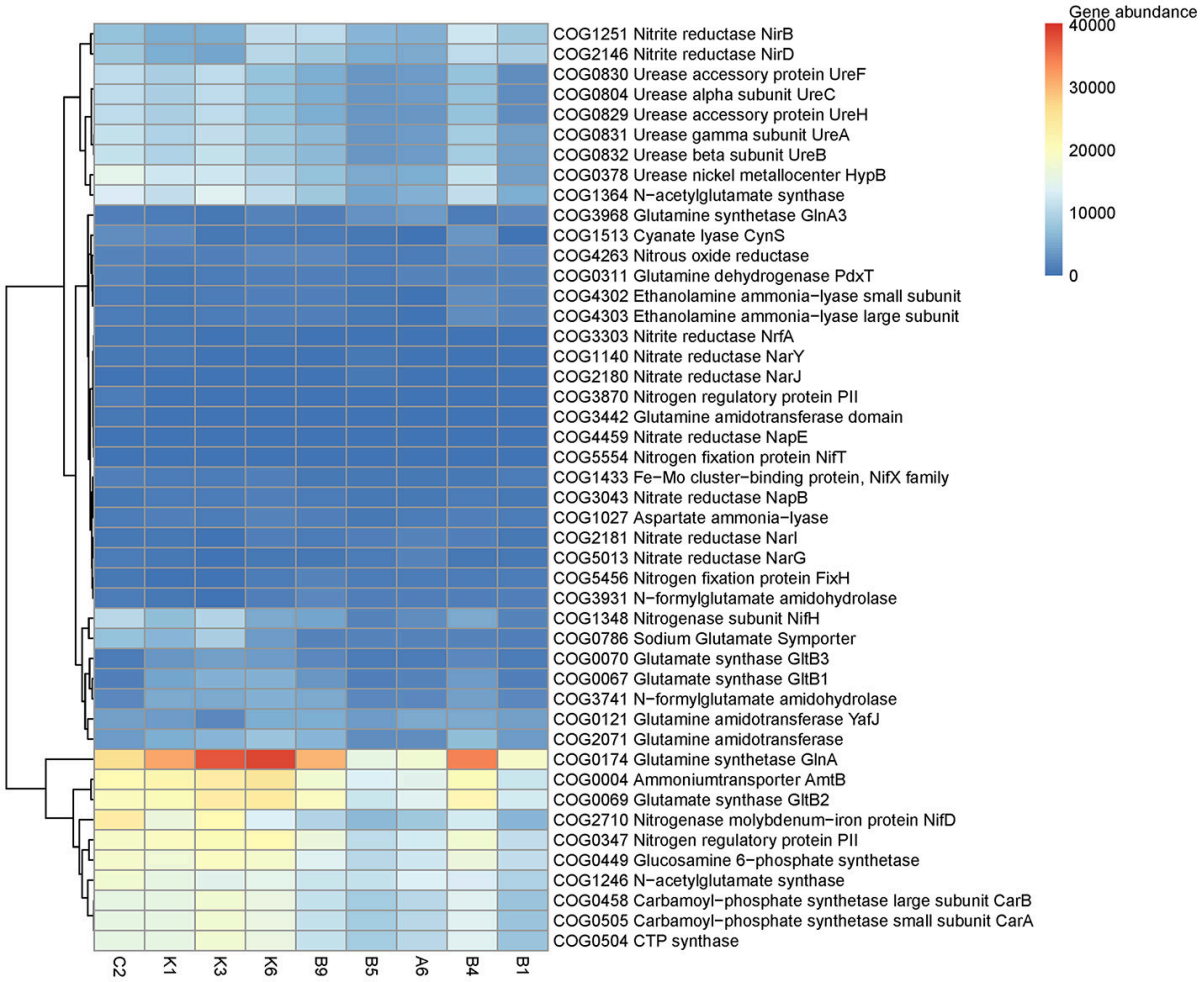
Abundances of COG functions related to N utilization using PICRUSt on the OTUs derived from the 16S rRNA analysis.

The predicted abundances of COG orthologs (COGs) assigned to the “transporters,” “inorganic nitrogen, urea, and amino acids” metabolisms showed clear variations of N utilization strategies in response to different N availabilities among sampling stations (Figure 7). The distributions of COGs assigned to ammonium transporter (*AmtB*, COG0004), ammonium assimilation enzymes (*GlnA*, COG0174 and *GltB2*, COG0069) and various COGs associated with amino acid biosynthesis were the most abundant in the sampling stations while abundance of COGs assigned to urease (*UreABCFH*, COG0829-COG0832, COG0804)) was high in the stations C2, K1, K3, K6, and B4. Interestingly, COGs related to N_2_ fixation (*NifH*, COG1348) and *NifD*, COG2710) presented high abundances in the N-deficient regions. Insignificant variations were observed regarding nitrate reductase and ammonia-lyase (Figure 7).

## Discussion

### Bacterial diversity in the NWPO

The northwestern Pacific gyre is one of the most oligotrophic oceans on Earth, experiencing widespread nutrient stress regarding N and phosphorus as well as great temperature differences (>10°) because of the mutual influence of the Kuroshio and Oyashio currents. The powerful Kuroshio-Current flows from the Philippines to southern Japan and is characterized by high diversity of phytoplankton along the current as the latitude increase (Ma et al., 2015). In contrast, the cold Oyashio current, originating from the Bering Strait, is characterized by high nutrient level (Saito et al., 2002). Thus, it provides a good model to study microbial structure and their adaptive mechanisms to ambient nutrient variations. In our study, both the bacterial diversity, including N fixing microorganisms, and the key N utilization gene expressions were investigated. Consistent with previous studies (Brown et al., 2009; Suh et al., 2014), the *Alpha-* and *Gammaproteobacteria* were ubiquitous in all stations, followed by the *Cyanobacteria*, which presented much higher abundances in the N-deficient stations K1 and K3 (Figure 2A). The alphaproteobacterial clade *SAR11* and gammaproteobacterial clade *SAR86* are key species surviving in the N-deficient South and North Pacific oceans (Eiler et al., 2009; West et al., 2016). Our study indicated that the *SAR11*clade exhibited relatively high abundance in the N-deficient transect K (Figure 2B), whereas they accounted for only a small proportion (<5%) of the total 16s rRNA gene sequences. Distributions of *SAR86* and *Flavobacteria* (*NS4, NS5, and NS9 group*) presented fluctuating patterns, which were similar to the microbial community in the eastern Pacific Ocean (Dupont et al., 2014). Our survey area covered the Kuroshio-Oyashio confluence region, the widespread distribution patterns of *SAR86* and *Flavobacteria* might result from the intense mixing of the prokaryotic community brought by circulation of these currents as surveyed by Severin et al. (2016). Recently, Li et al. (2017) reports that the Kuroshio intrusion impacts the picoplankton abundance and community structure in the northeastern South China Sea (SCS).

The relative abundance of the cyanobacterial genus *Prochlorococcus* was the highest in the surface water of the N-deficient station K3, and *Prochlorococcus* combined with the gammaproteobacterial *Pseudoalteromonas* dominated the bacterial community in the N-deficient station K1. Species of *Prochlorococcus* are widely distributed in the oligotrophic oceans such as the SCS (Wang et al., 2011), South Atlantic Ocean (Morris et al., 2010) and central Pacific Ocean (Saito et al., 2014), owing to their strong adaptive ability to low ambient nutrients (Garcia-Fernandez et al., 2004). *Pseudoalteromonas* is generally associated with marine eukaryotes and displays anti-bacterial activity. The active production of substrates by *Pseudoalteromonas* can out-compete themselves and their hosts in the colonization of surface waters and the acquisition of nutrients (Holmstrom and Kjelleberg, 1999). However, this hypothesis requires further physiological evidence.

*Synechococcus* was widely distributed among the sampling area with high abundance in the Kuroshio-Oyashio confluence region. Previous study also reports that *Synechococcus* is mainly distributed in the surface waters adjacent to the cyclonic eddy center induced by Kuroshio intrusionin the SCS, where high concentration of dissolved reactive phosphorus is detected (Li et al., 2017). Though *Synechococcus* is virtually ubiquitous in all marine environments, it is much more abundant in nutrient-rich areas (Partensky et al., 1999). The nutrient-replete Kuroshio-Oyashio confluence region might provide sufficient nutrients for the growth of *Synechococcus*.

*Cyanobacteria* are not only the important primary producers but also responsible for N fixation in the oligotrophic Pacific Ocean (Montoya et al., 2004; Church et al., 2005; Moisander et al., 2010). In our study, *UCYN-A* dominated the diazotrophic community in the N-deficient stations K1, K3, and K6 (Figure 3B), which was in agreement with a previous study that *UCYN-A* cells are distributed only in the oligotrophic region of the North Atlantic Ocean (Krupke et al., 2014). At present, at least three lineages of *UCYN-A* are detected in the ocean and *UCYN-A*2 is thought to be easier adapted to coastal waters (Bombar et al., 2014). Owing to the limitation of the method, we failed to obtain information of the *UCYN-A* lineage.

The unicellular N_2_-fixing *Proteobacteria* were the second most widespread group and mainly localized to N-sufficient stations. The relative abundance of the gammaproteobacterial *Pseudomonas* was high in the coastal station C2 (Figure 3), which is separated from other stations by low temperature, thus suggesting a better adaptability of *Pseudomonas* to lower temperature as indicated by Suyal et al. (2014).

Environmental variables play important roles in shaping bacterial diversity and community structure (Holmstrom and Kjelleberg, 1999). In our study, the results of mental test and correlation analysis showed that temperature was the main factor regulating the bacterial community structure and between-habitat diversity (Tables 2, 3; Table S2), which was in agreement with previous studies in different regions of oceans (Pomeroy and Wiebe, 2001; Fuhrman et al., 2008). Whereas, concentration of NO_X_-N was the main factor affecting the structure and diversity of non-filamentous diazotrophic community when temperature was not a limiting factor (Figure 3; Tables 2, 3). As expected, the distributions of unicellular N_2_-fixing *Proteobacteria* and *Cyanobacteria* presented opposite trends with regard to concentrations of NO_X_-N, suggesting that nutrient availability was the most important factor determining the non-filamentous diazotrophic community composition (Figure 5). In the Atlantic Ocean, non-filamentous nifH sequences cover a broader temperature range while filamentous cyanobacterial nifH sequences are adapted to narrow temperature (Langlois et al., 2005).

Likewise, it should be pointed out that 2% gene sequences were unclassified, particularly in the confluence station B4, owing to the lack of sufficient database coverage for taxon-specific genomes. However, our results still indicated that temperature and nutrient availability shaped microbial communities and the interaction of ocean currents influenced some bacterial distributions in the NWPO when compared with other ocean regions.

### Nitrogen utilization strategies of *Cyanobacteria*

Understanding the distribution and N utilization strategies of marine microorganisms in relation to nutrient availability is a key problem in microbial oceanography. The availability of N is important in regulating biological productivity in marine environments and biological N_2_ fixation has a critical role in supporting oceanic new production (Zehr and Ward, 2002). A previous study reports a link between new production in oligotrophic waters and unicellular diazotrophs (Montoya et al., 2004). In our study, the predicted nitorgenase *nifH* and *nifD* were more abundant in the N-deficient regions where high abundance of unicellular diazotrophic *cyanobacteria* was detected, indicating a high contribution of N_2_ fixationby unicellular *cyanobacteria* in the N-deficient regions.

The cyanobacterial genera *Prochlorococcus* and *Synechococcus* dominated prokaryotic communities in the oligotrophic NWPO surface waters, and *Prochlorococcus* genes are among the most highly expressed in the oligotrophic Pacific Ocean (Frias-Lopez et al., 2008). ${\text{NH}}_{4}^{+}$ is an important regenerated N source that can be utilized by both *Prochlorococcus* and *Synechococcus* (Lindell et al., 2002). Our study showed that expressions of the *amt1* gene were high in the surface waters compared to the DCM layers and far higher than that of *NarB* and *urtA* (Figures 6A–C.) The PICRUSt result presented here fitted the trend: COGs assigned to ammonium transporter and two key ammonium assimilation enzymes (*GlnA* and *GtlB*), were the most abundant in surface waters of sampling regions (Figure 7), suggesting that ${\text{NH}}_{4}^{+}$ was still the most favorable N source for microorganisms. However, ${\text{NH}}_{4}^{+}$ is quickly converted in surface waters (Tolonen et al., 2006), thus cells highly expressed ammonium transporter and assimilation enzymes to acquire nutrients.

Interestingly, *narB* gene transcripts was substantially lower than that of *amt1* and *urtA* in the N-deficient surface waters despite low concentrations of DIN, but the gene expression was enhanced in the DCM layers of the N-sufficient stations A6 and B1 (Figures 6B, D). Meanwhile, PICRUSt results also showed that the abundance of nitrate reductases was relatively lower than those genes involved in metabolisms of ammonium, urea and amino acids (Figure 7).The low expression of the *narB* gene was not clear since subtropical surface waters experienced widespread N-deficient stress, which might drive cells to synthesize more metabolic enzymes to meet nutrient requirements. We proposed that the *narB* gene could be substrate-induced and short of relevant regulatory binding sites. It is reported that the *narB* gene in *Synechococcus* is not up-regulated under N-starvation but its transcripts increase following nitrate addition (Su et al., 2006; Paerl et al., 2012). High concentrations of nutrients (${\text{NO}}_{3}^{-}$, ${\text{NO}}_{2}^{-}$, ${\text{NH}}_{4}^{+}$, and ${\text{PO}}_{4}^{3-}$) in the DCM layers might generate amounts of substrate, and hence increased the expression of the *narB* gene as shown in Figure 6B. Recently, the widespread occurrence of nitrate assimilating gene, *narB*, has been discovered in uncultured *Prochlorococcus* from marine waters (Martiny et al., 2009), but we failed to amplify the *Prochlorococcus narB* gene products following Martiny et al. (2009) owing to the species specificity, so we used the *Synechococcus narB* genes instead.

Generally, *Cyanobacteria* can utilize not only DIN, such as ${\text{NO}}_{3}^{-}$, ${\text{NO}}_{2}^{-}$, and ${\text{NH}}_{4}^{+}$, but also DON containing reduced N nutrients (Flombaum et al., 2013). Urea, occurring at nanomolar level in the open ocean, dominates DON pools and provides nearly half of the phytoplankton N uptake annually (Connelly et al., 2014). In different marine ecosystems, *Cyanobacteria* possess diverse N utilization strategies and increase the absorption rates of organic N compounds in N-deficient regions (Hewson et al., 2010). Our results showed that both the COGs assigned to urease and expressions of *urtA* were very high in N-deficient surface waters (Figures 6C,D, 7), indicating that urea-fueled nitrification by *Cyanobacteria* helped them survive in the fierce nutrient competition conditions, which is in accordance with the studies of the central Pacific Ocean and the SCS, where urea transporter is abundant in the oligotrophic surface water layer (Wang et al., 2011; Saito et al., 2014). However, no OCGs assigned to urea transporter were found but abundant unclassified ABC transporters were predicted, suggesting that further studies of metagenomics or metaproteomics are necessary to unveil N utilization strategies of bacteria.

In general, *Prochlorococcus* and *Synechococcus* inhabit diverse environments and their N utilization strategies differ in response to ambient N availability, so the gene expression patterns based on the genus specific primers targeting *urtA* and *amt1* would be more precise to illustrate the *in situ* N utilization strategies. However, alignments of *urtA* or *amt1* genes among *Prochlorococcus* and *Synechococcus* strains present high similarity, and *urtA* sequences reach more than 80% similarity among 24 *Prochlorococcus* strains and 9 *Synechococcus* strains (data were retrieved from the Cyanobacterial Knowledge Base). Similar result is also found in the alignment of *amt1* genes. Thus, it is difficult to design the genus specific primers to separate *Prochlorococcus* from *Synechococcus*, which impedes our understanding of N utilization strategies of these two genera. In the future, sequencing full-length *urtA* and *amt1* genes and corresponding complementary DNA are necessary to better understand specific N utilization strategies of *Prochlorococcus* and *Synechococcus*.

## Conclusions

This study provided a first glance at the bacterial diversity, diazotrophic diversity and their N utilization strategies in different N regimes of the NWPO. Bacterial diversity presented distinct geographic features while the *Cyanobacteria* contributed both primary productivity and a significant fraction of new production in the N-deficient oceans. Temperature was the main factor affecting structure, diversity, and distribution of bacterial community while concentration of NO_X_-N played key roles in shaping the structure and diversity of diazotrophic community. Among the predominant cyanobacterial ecotypes, N utilization strategies shifted with the availability of ambient N. The expression pattern of *urtA* transcripts and the distribution of predicted urease in surface waters highlighted the ecological significance of urea in the oligotrophic ocean, and the shift from nitrate to reduced nitrogen in *Cyanobacteria* might be the key to their dominance in the N-deficient oceanic waters. In the future, comprehensive studies are necessary to investigate microbial diversity and their N utilization strategies in diverse N regimes of the oligotrophic ocean using metagenomic and metaproteomic approaches, to further our understanding of the bacterially mediated N cycle in the ocean.

## Author contributions

D-ZW and Y-YL: designed the study and wrote the paper; Y-YL and X-HC: performed the experiment; P-FW, D-XL, and L-FK: contributed to sample collection; Z-XX, S-JK and LL: contributed to data analysis.

### Conflict of interest statement

The authors declare that the research was conducted in the absence of any commercial or financial relationships that could be construed as a potential conflict of interest.
