# Supplementary figures and images for: Bacterial Diversity and Nitrogen Utilization Strategies in the Upper Layer of the Northwestern Pacific Ocean
Source: Front Microbiol. 2018 Apr 25;9:797. doi: 10.3389/fmicb.2018.00797 (PMC5996900; doi:10.3389/fmicb.2018.00797)

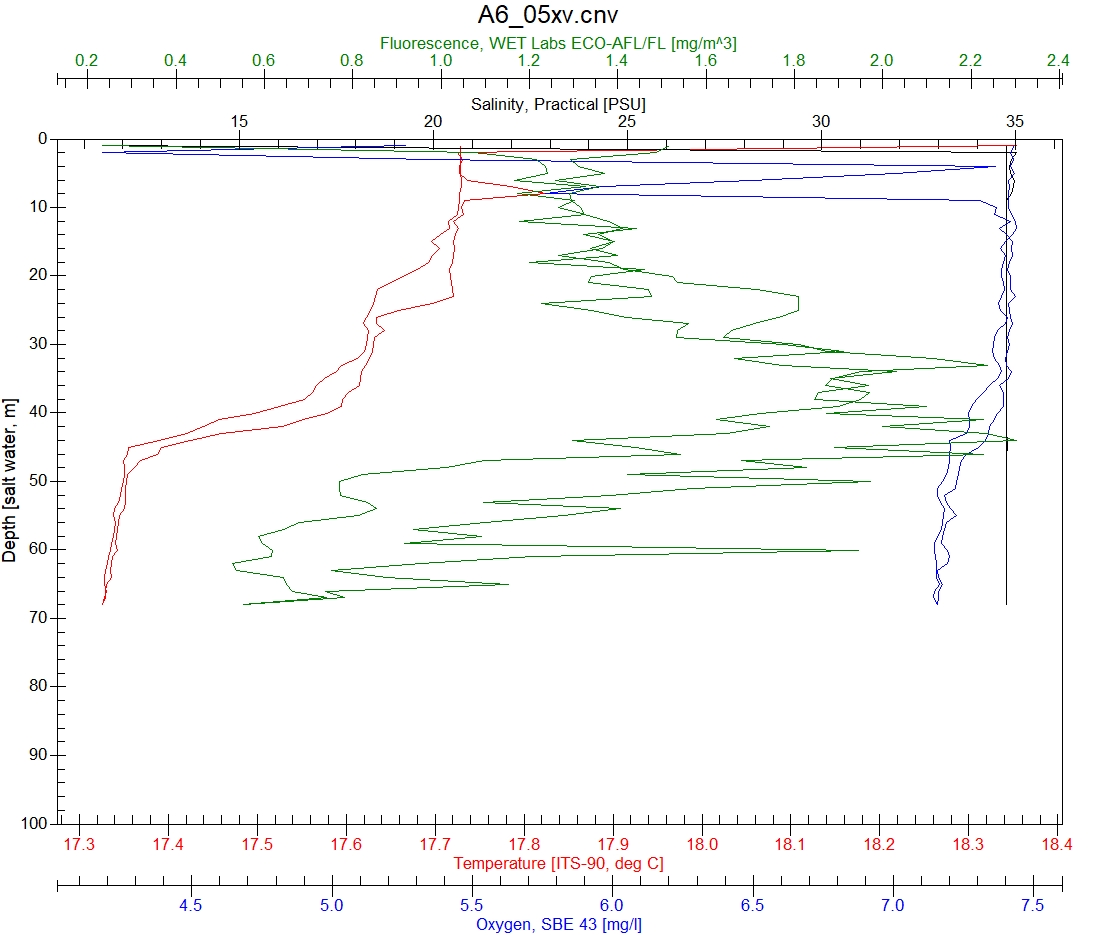

Supplement: Supplementary file 2 [file Data_Sheet_2.ZIP › CTD data/A6.jpg]

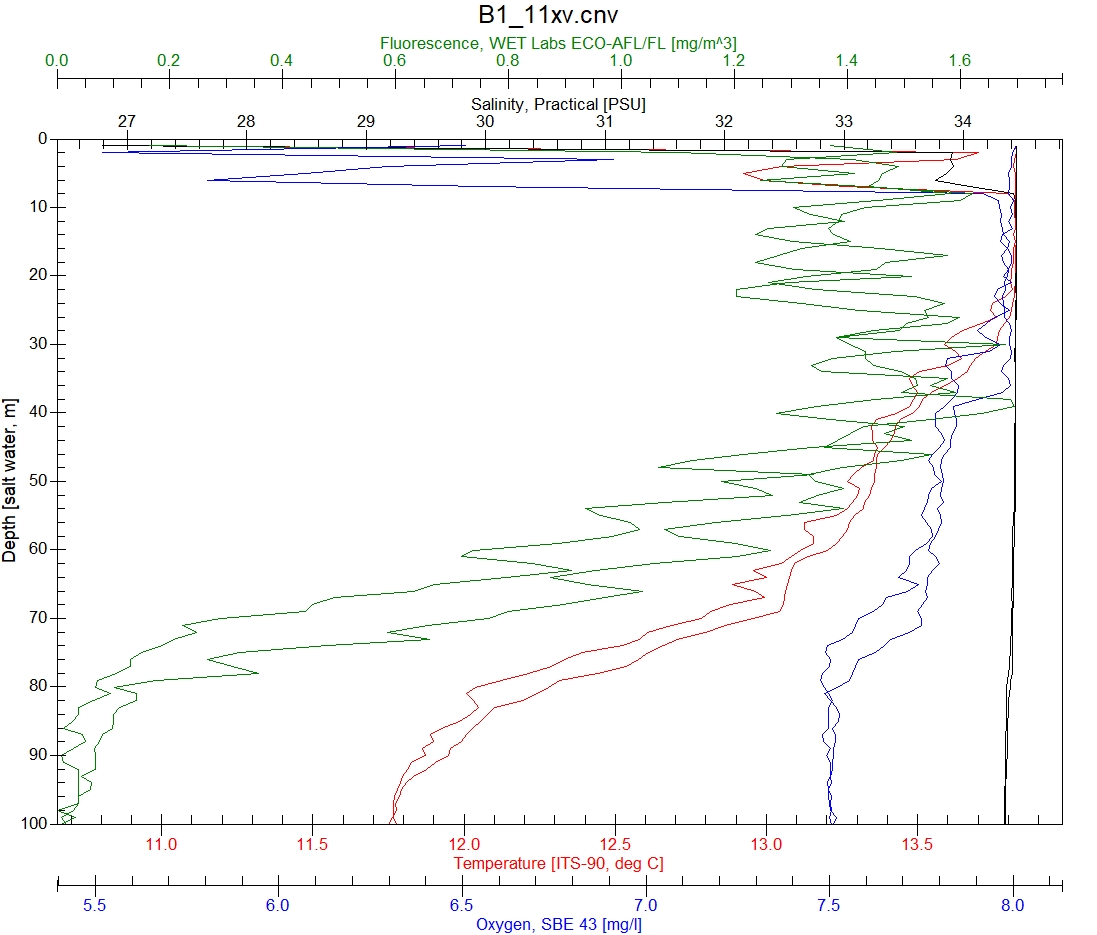

Supplement: Supplementary file 2 [file Data_Sheet_2.ZIP › CTD data/B1.jpg]

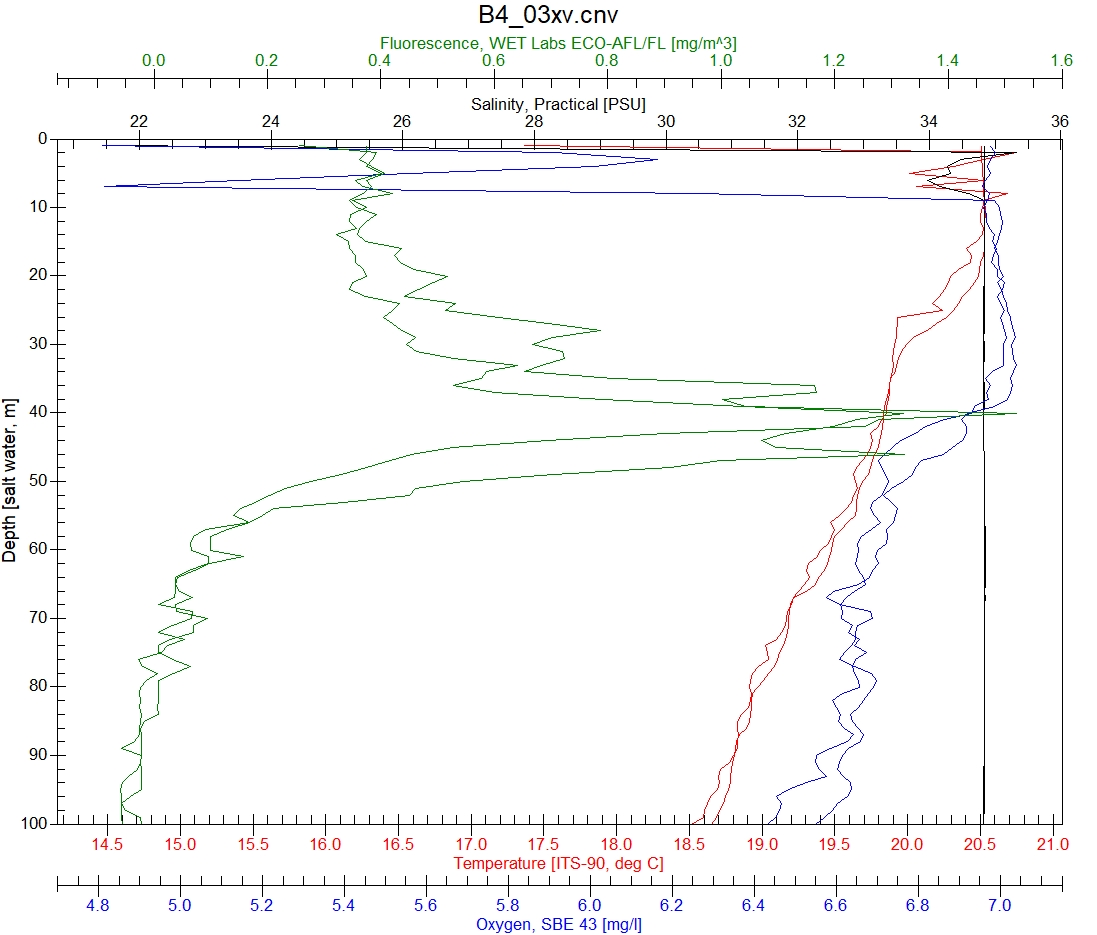

Supplement: Supplementary file 2 [file Data_Sheet_2.ZIP › CTD data/B4.jpg]

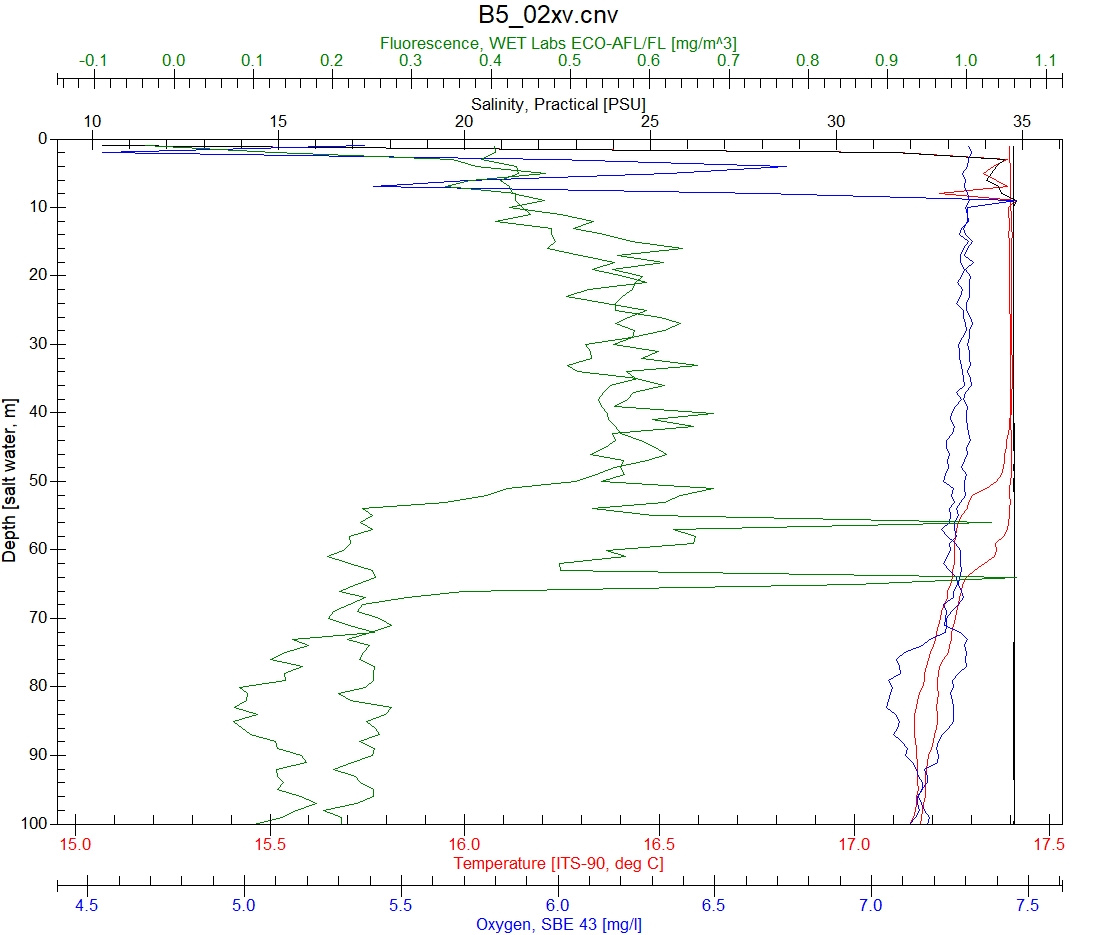

Supplement: Supplementary file 2 [file Data_Sheet_2.ZIP › CTD data/B5.jpg]

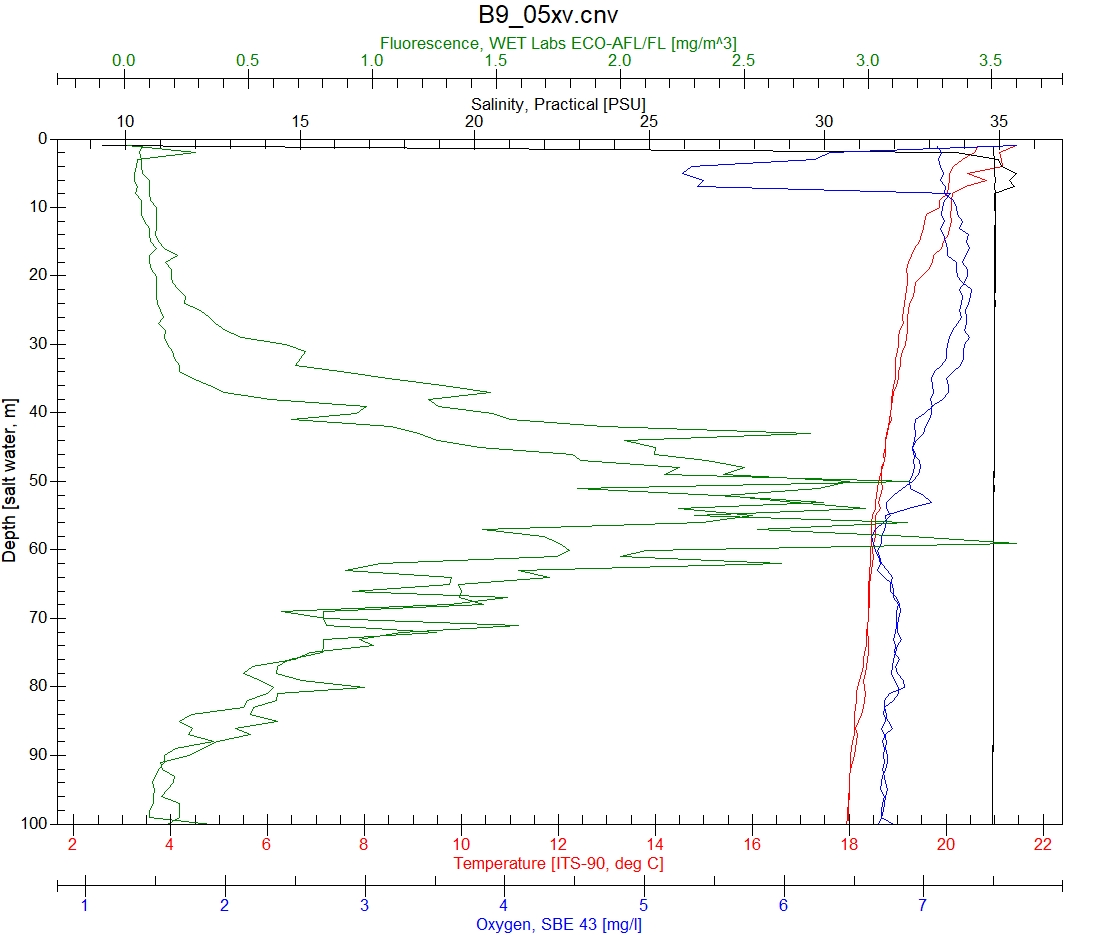

Supplement: Supplementary file 2 [file Data_Sheet_2.ZIP › CTD data/B9.jpg]

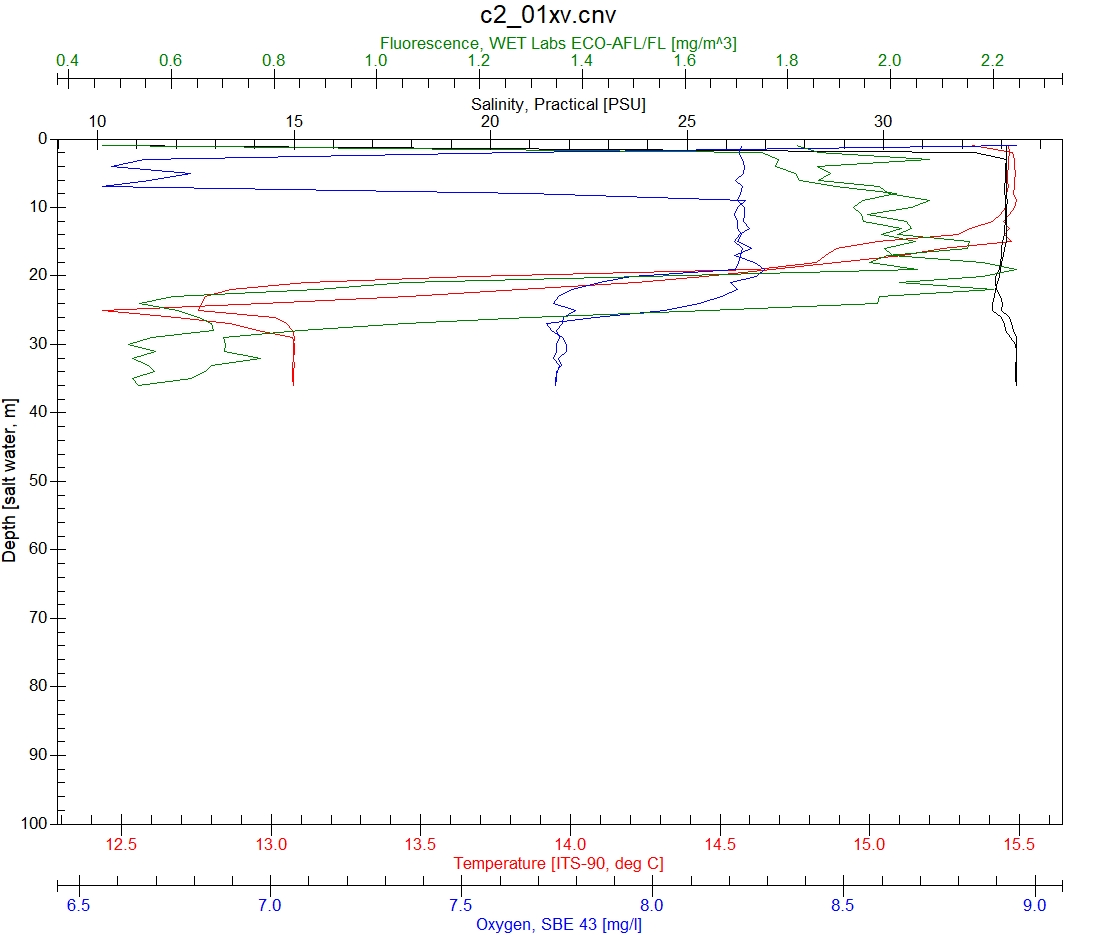

Supplement: Supplementary file 2 [file Data_Sheet_2.ZIP › CTD data/C2.jpg]

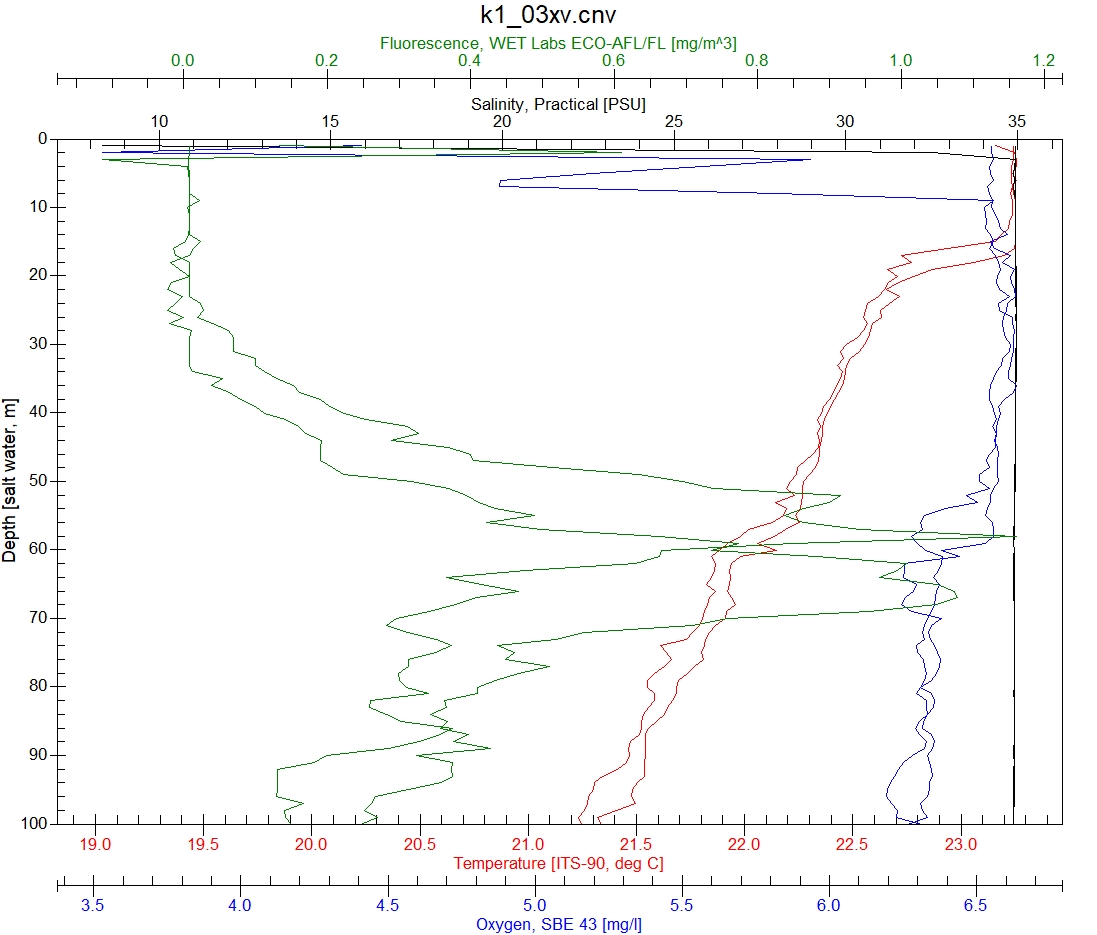

Supplement: Supplementary file 2 [file Data_Sheet_2.ZIP › CTD data/K1.jpg]

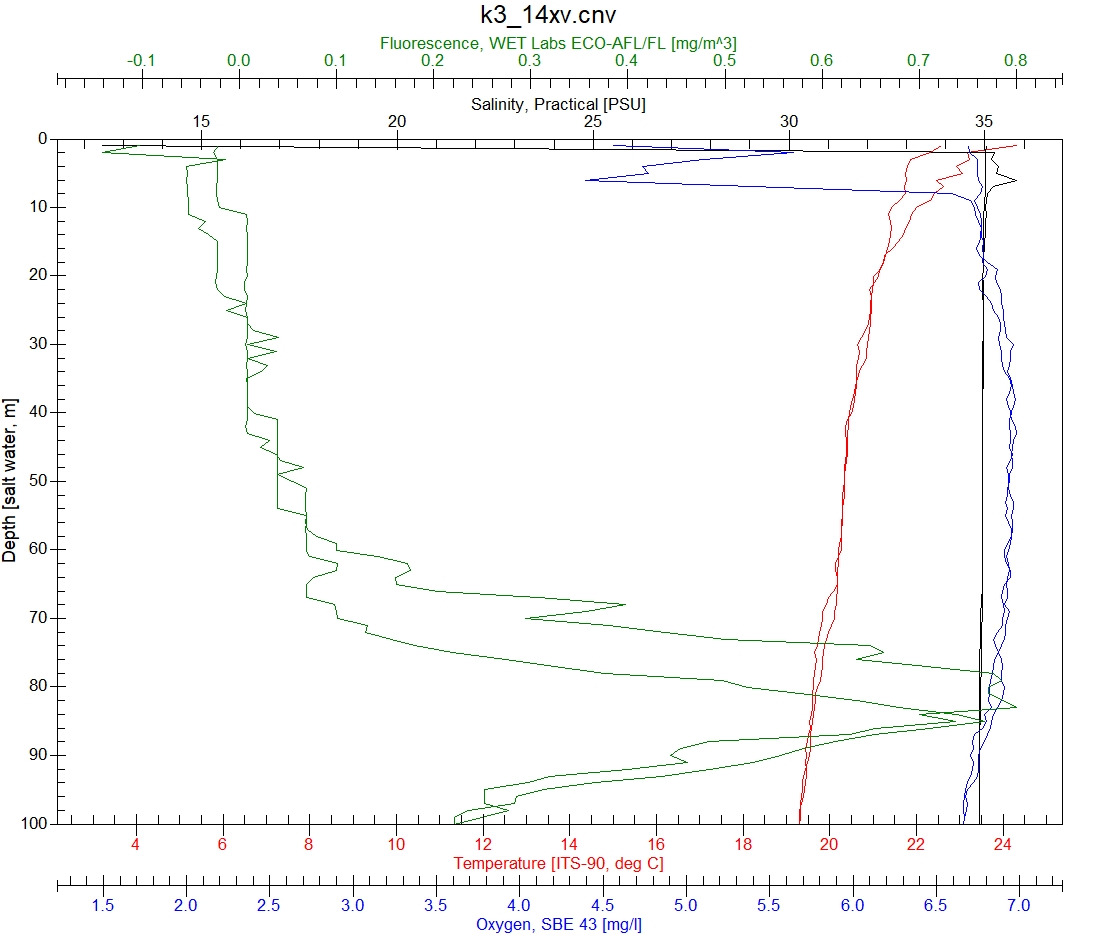

Supplement: Supplementary file 2 [file Data_Sheet_2.ZIP › CTD data/K3.jpg]

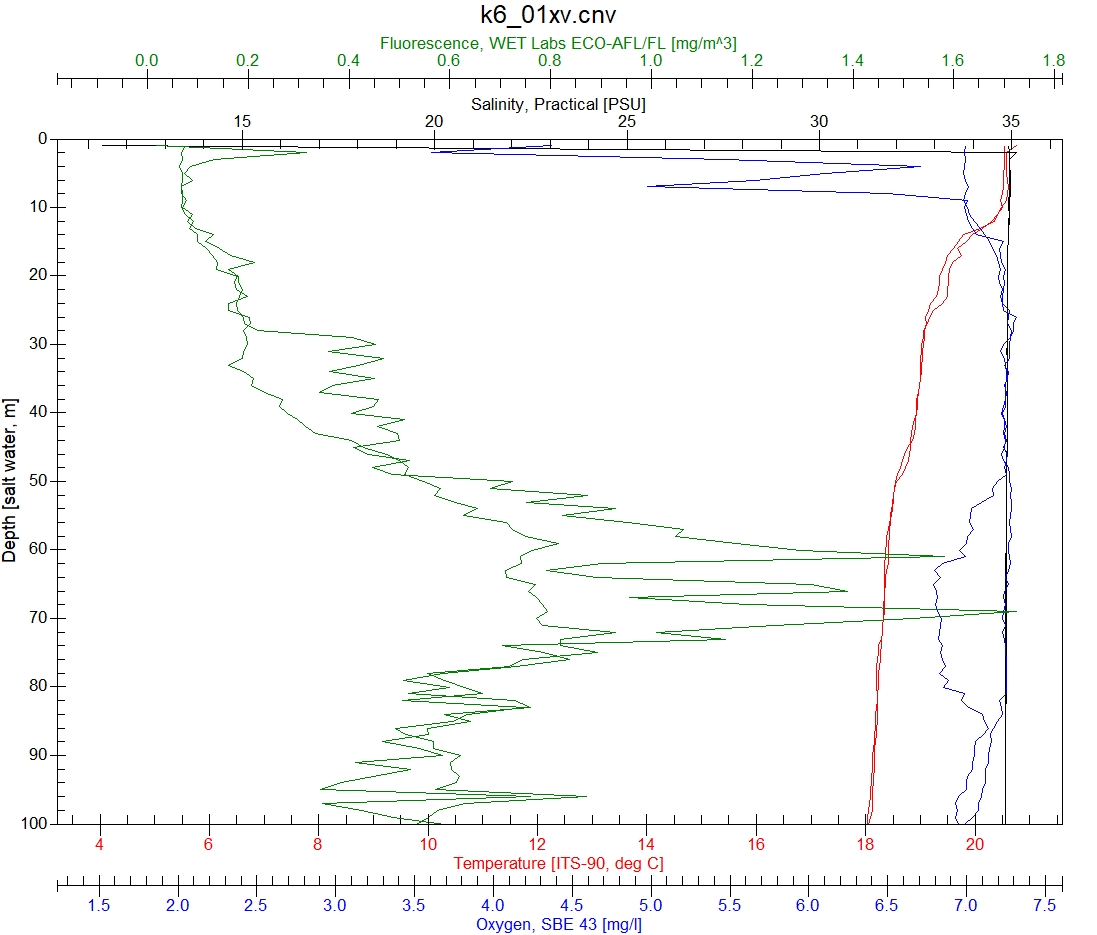

Supplement: Supplementary file 2 [file Data_Sheet_2.ZIP › CTD data/K6.jpg]
